# A Case of Vaping-Associated Candida and Herpes Simplex Virus (HSV) Co-infection Causing Esophagitis in an Immunocompetent Patient

**DOI:** 10.7759/cureus.60710

**Published:** 2024-05-20

**Authors:** Sonia Alicea, Natalia Tejada, Jorge Restrepo, Amar Mandalia

**Affiliations:** 1 Internal Medicine, University of Central Florida/HCA Healthcare Graduate Medical Education (GME), Orlando, USA; 2 Internal Medicine, University of Central Florida, Orlando Veterans Affairs Medical Center, Orlando, USA; 3 Gastroenterology, Orlando Veterans Affairs Medical Center, Orlando, USA

**Keywords:** vaping, candida, hsv, immunocompetent, co-infection, gerd, electronic cigarette, smoking, esophagitis, effects of vaping

## Abstract

Electronic cigarettes (e-cigarettes) and vaping have gained popularity in the last two to five years as an alternative way of consuming nicotine, as well as tetrahydrocannabinol (THC), particularly in the younger population. Vaping/e-cigarettes heat nicotine/THC and other chemical components to create the vapor to be inhaled, which increases the risk of mucosal infection and esophagitis. Although tobacco smoking has been extensively studied and known to affect the oral cavity and esophagus, the effect of vaping is yet to be well-studied. We report a case of odynophagia secondary to esophageal candidiasis, herpes simplex virus (HSV) esophagitis, and reflux esophagitis associated with vaping.

## Introduction

Tobacco smoking affects the oral cavity, with an increased risk of mucosal infections, including candidiasis. E-cigarettes/vaping have gained popularity in the last two to five years as an alternative way of consuming nicotine and tetrahydrocannabinol (THC), particularly in the younger population. In an annual, nationally representative survey reported by the National Health Interview Survey (NHIS) in 2021, the prevalence of e-cigarette use was 4.5%, increasing from 3.7% to 4.5% in comparison with the previous year [1].

Vaping/e-cigarettes heat nicotine/THC and other chemical components to create the vapor to be inhaled. It is important to note that chemical products, fine particulate matter (PM), or ultrafine particles (UFPs) present in e-cigarettes can vary upon different devices [2]. There is evidence that e-cigarettes may produce PM concentrations approximately 45 times higher and UFP concentrations about 20 times higher than the recommended by the World Health Organization (WHO) [3]. These particles have well-established negative effects on the respiratory and cardiovascular systems. Additionally, the heating temperatures of the e-cigarette substance release metal particles such as copper, nickel, and silver from the device into the bloodstream through the lungs [2,4]. Exposure to metals is known to have potential effects on the overall health of exposed individuals.

Although tobacco smoking has been extensively studied and known to affect the oral cavity and esophagus, the effect of vaping is yet to be well-studied. Here, we present a case of severe candida and herpes simplex virus (HSV) esophagitis associated with vaping, which, to our knowledge, is the first reported case.

## Case presentation

**Figure 1:**
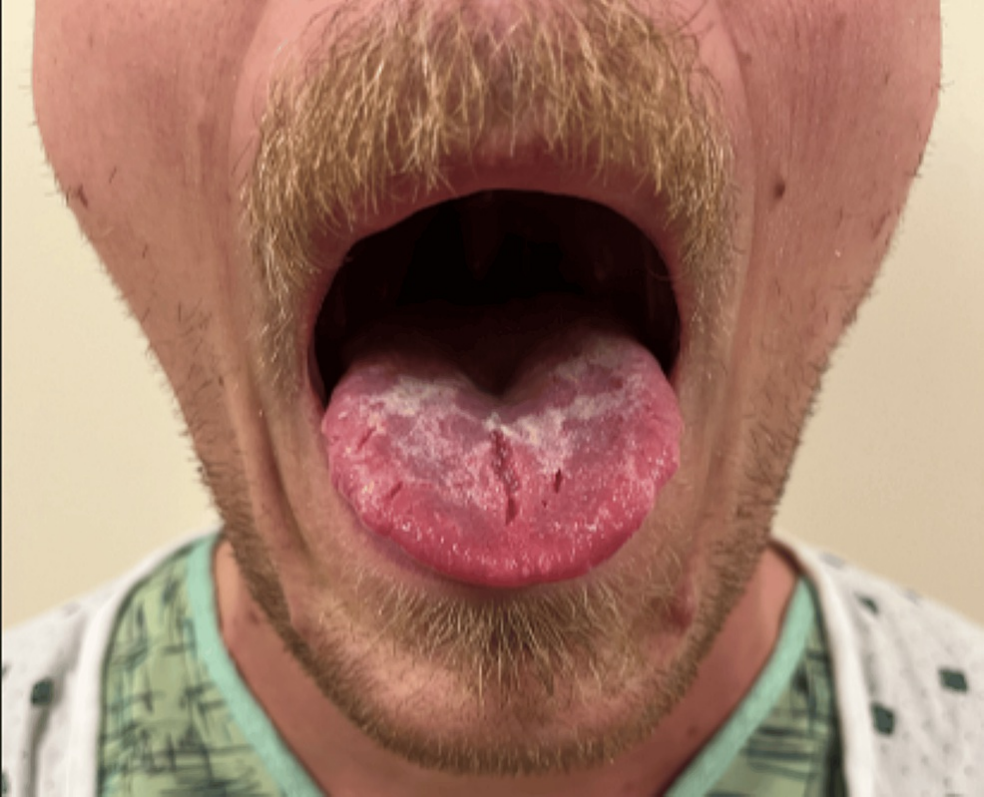
Non-scrapable thick, whitish plaques on the tongue suggestive of oral candidiasis.

A 30-year-old man, with a history of mild gastroesophageal reflux disease (GERD), reporting only intermittent, none progressive, one to two episodes per month that are well-controlled on famotidine as needed, as well as current heavy vaping, presented to the hospital with progressive acute moderate odynophagia associated with intermittent sharp chest pain, malaise, and subjective fever. He denied alcohol use, non-steroidal anti-inflammatory use, or tobacco smoking, but reported daily heavy vaping for one to two years, with frequent vape sharing with friends. The patient was afebrile, with non-scrapable thick, whitish plaques on the anterior tongue (Figure 1), and a non-tender abdomen on physical examination.

Computed tomography (CT) of the chest with intravenous contrast was done, and results demonstrated enlarged lymph nodes at the subcarinal, bilateral tracheal, right hilar, and gastrohepatic areas; no esophageal abnormalities were reported. The patient was started on nystatin swish and swallow empirically.

**Figure 2:**
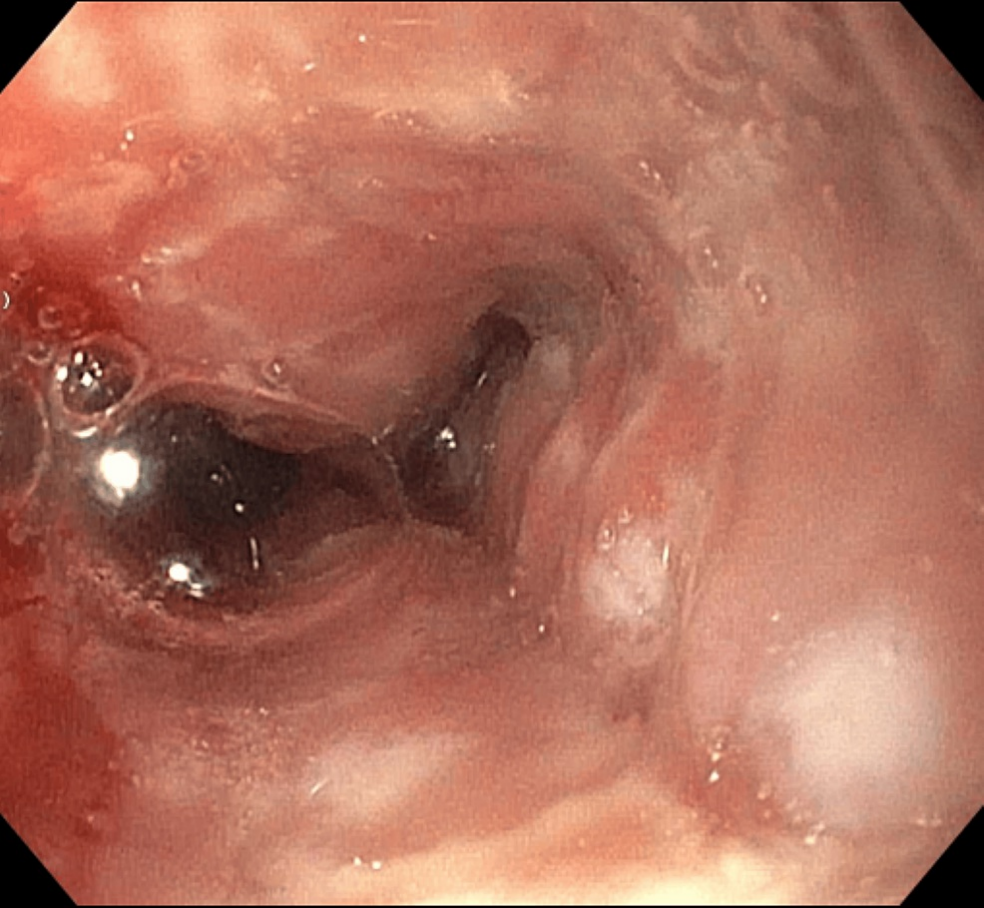
Esophagus during EGD showing severe monilial esophagitis in the entire esophagus.

**Figure 3:**
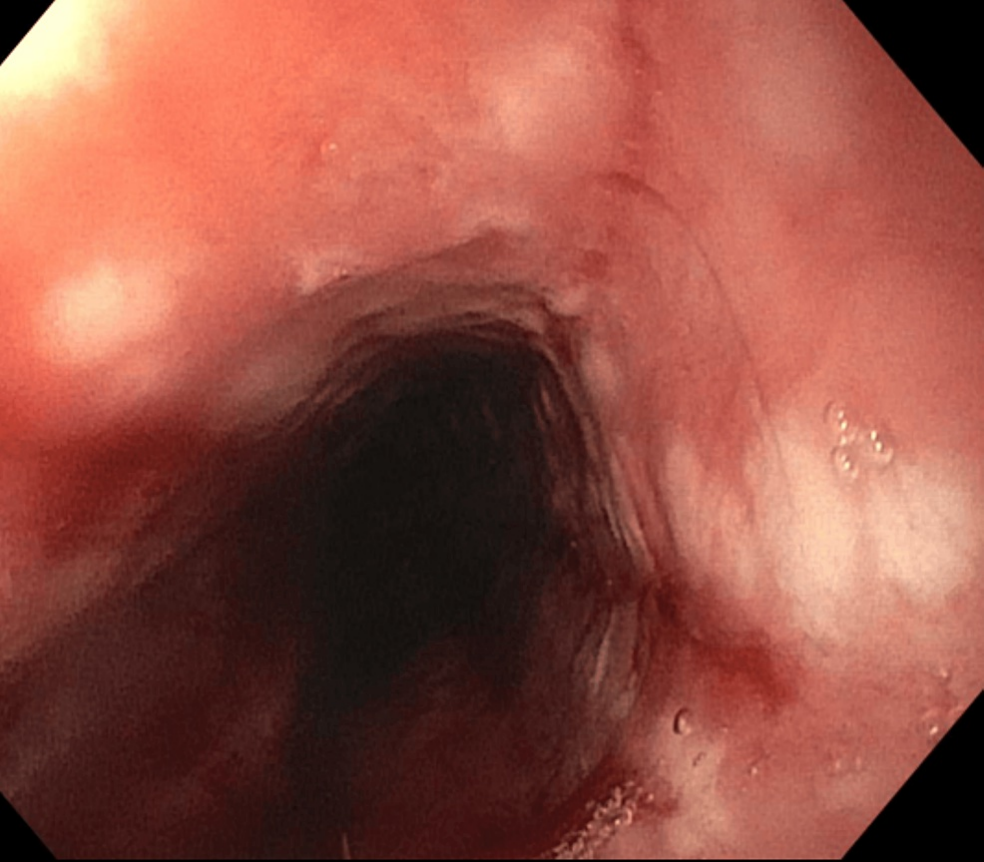
Esophagus during EGD showing severe monilial esophagitis in the entire esophagus. EGD: esophagogastroduodenoscopy

**Figure 4:**
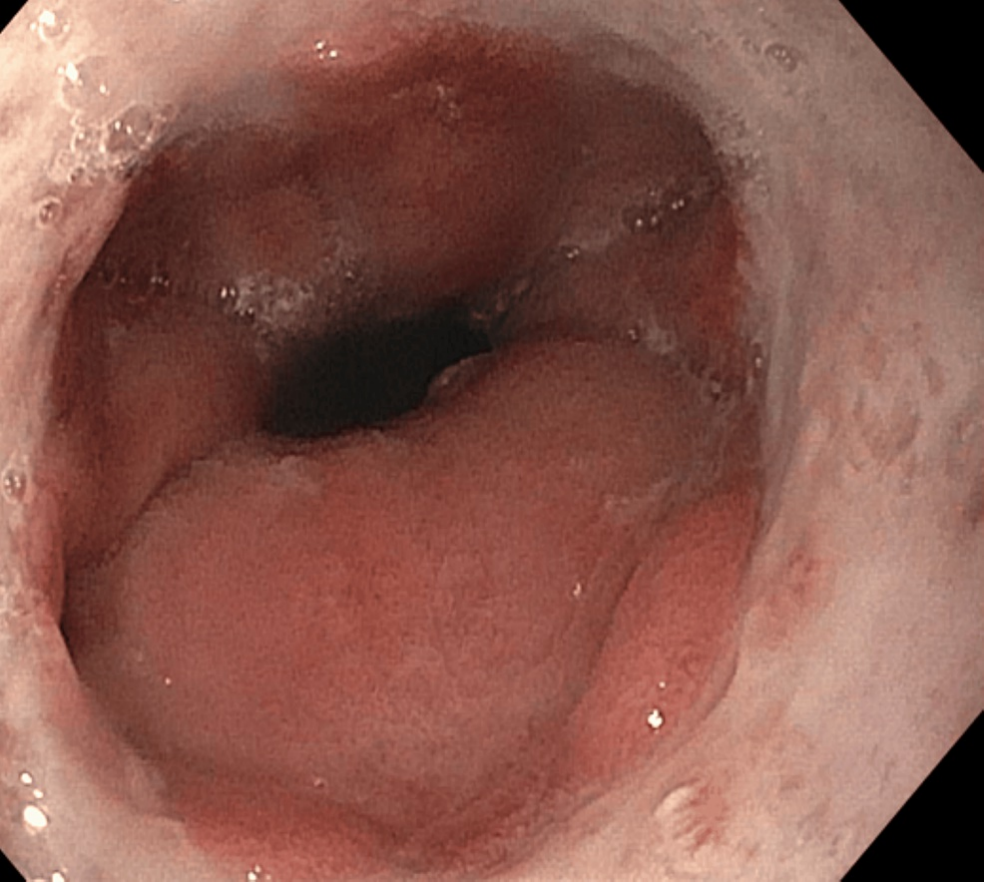
Distal esophagus during EGD showing Los Angeles Grade D reflux esophagitis with Z-line irregularities 40 cm from the incisors, also showing a cratered circumferential distal esophageal ulcer. EGD: esophagogastroduodenoscopy

Gastroenterology was consulted, and esophagogastroduodenoscopy (EGD) was performed. The results showed severe monilial esophagitis, distal esophageal ulcer, Los Angeles Grade D reflux esophagitis with Z-line irregularities (40 cm from the incisors), and 3 cm hiatal hernia (Figures 2-4).

The histopathologic analysis demonstrated a negative periodic acid-Schiff (PAS) stain for fungal elements, negative Helicobacter pylori, severely active cardio-esophagitis with ulceration and intestinal metaplasia/Barrett’s esophagus, and mid-esophagus herpes simplex virus (HSV) esophagitis. HIV serology was negative. The patient was treated with analgesics, pantoprazole, and fluconazole and discharged on oral pantoprazole and oral fluconazole daily for 14 days. Lifestyle modifications were recommended, including avoidance of vaping/e-cigarette use. He was also scheduled by the gastroenterology team for a repeat EGD in eight weeks. On a two-week telehealth follow-up, the patient reported that his odynophagia had completely resolved. HSV treatment was not recommended since the patient was immunocompetent and HSV infection had likely self-resolved.

## Discussion

The long-term complications of vaping are currently unknown; however, its short-term complications include well-known acute lung injury [5]. Vaping-associated non-infectious esophagitis was described in one case report; to our knowledge, it might be one of the only cases reported [6]. Studies have demonstrated that nicotine from different sources may affect Candida albicans growth and expression of virulence-related genes [7]. E-cigarettes produce free radicals that cause direct mucosal injury and affect the oral cavity’s innate immune system, increasing the risk of oral infections [8]. In addition, one of the mechanisms of injury due to vaping could be caused by the effects of nicotine, as well as THC, which can be present in the vaping substrate. Like nicotine, THC has also been shown to influence the regulation of transient lower esophageal sphincter relaxations and lower esophageal pressure, increasing the risk of GERD [9]. The effect of smoking on the progression of GERD has been previously described [10,11]. Nonetheless, the effect of vaping on GERD requires further investigation.

We presented a case of odynophagia secondary to esophageal candidiasis, HSV esophagitis, and reflux esophagitis associated with vaping. As described before, the patient had baseline mild GERD, with only one to two episodes per month, for which the patient did not report worsening of symptoms in the last month before he developed odynophagia. This part of the medical history described, as well as the changes found on the EGD, makes reflux esophagitis less likely a major component causing this patient's odynophagia. Additionally, the patient's symptoms improved soon after being treated with antifungal medication, making monilial esophagitis likely the major cause of his odynophagia. Furthermore, the patient reported daily heavy vaping for one to two years, with frequent vapes sharing with friends. Vape sharing could have increased the risk of exposure to candida and HSV, as well as the susceptibility to it. This together with the changes in the oral microbiome that come with nicotine/THC exposure to vapors, contributed to the patient's risk of esophagitis. Even though the PAS stain was negative, this represents most likely a false-negative result given the significant monilial esophagitis seen on EGD. In this case, the patient was treated with nystatin swish and swallow empirically before EGD was performed, which could have contributed to the PAS stain resulting in a false negative.

## Conclusions

This case report emphasizes the importance of suspecting vaping-associated esophagitis in patients with odynophagia and current vaping/e-cigarettes use. Furthermore, it helps healthcare providers to recognize the importance of screening for vaping/e-cigarette use in patients with new-onset, persistent, or progressive GERD symptoms. Healthcare providers’ awareness of these risks is important for proper screening and counseling of gastrointestinal complications of vaping. Due to the increasing prevalence of e-cigarette use, further investigation of the potential complications of vaping/e-cigarettes and their implications in the healthcare system is of utmost importance. Additional studies and case reports are warranted to further evaluate the long-term effects of vaping/e-cigarettes use.
